# The PRISMATIC project: protocol for a research programme on novel methods to improve reporting and peer review of systematic reviews of health evidence

**DOI:** 10.1186/s13643-023-02363-6

**Published:** 2023-10-13

**Authors:** Matthew J. Page, David Moher, Sue Brennan, Joanne E. McKenzie

**Affiliations:** 1 Methods in Evidence Synthesis Unit, School of Public Health and Preventive Medicine, Monash University, 553 St Kilda Road, Melbourne, VIC 3004 Australia; 2 Centre for Journalology, Clinical Epidemiology Program, Ottawa Hospital Research Institute, Ottawa, ON Canada; 3 School of Epidemiology and Public Health, Faculty of Medicine, University of Ottawa, Ottawa, ON Canada; 4 School of Public Health and Preventive Medicine, Monash University, Melbourne, VIC Australia

**Keywords:** Systematic reviews, Meta-analysis, Reporting, Transparency, Methodology

## Abstract

**Background:**

Incomplete reporting about what systematic reviewers did and what they found prevents users of the report from being able to fully interpret the findings and understand the limitations of the underlying evidence. Reporting guidelines such as the PRISMA statement and its extensions are designed to improve reporting. However, there are important inconsistencies across the various PRISMA reporting guidelines, which causes confusion and misinterpretation. Coupled with this, users might need to consult multiple guidelines to gain a full understanding of the guidance. Furthermore, the current passive strategy of implementing PRISMA has not fully brought about needed improvements in the completeness of systematic review reporting.

**Methods:**

The PRISMATIC (‘PRISMA, Technology, and Implementation to enhance reporting Completeness’) project aims to use novel methods to enable more efficient and effective translation of PRISMA reporting guidelines into practice. We will establish a working group who will develop a unified PRISMA statement that harmonises content across the main PRISMA guideline and several of its extensions. We will then develop a web application that generates a reporting template and checklist customised to the characteristics and methods of a systematic review (‘PRISMA-Web app’) and conduct a randomised trial to evaluate its impact on authors’ reporting. We will also develop a web application that helps peer reviewers appraise systematic review manuscripts (‘PRISMA-Peer app’) and conduct a diagnostic accuracy study to evaluate its impact on peer reviewers’ detection of incomplete reporting.

**Discussion:**

We anticipate the novel guidance and web-based apps developed throughout the project will substantively enhance the completeness of reporting of systematic reviews of health evidence, ultimately benefiting users who rely on systematic reviews to inform health care decision-making.

## Background

Systematic reviews serve many vital roles. They can provide syntheses of the state of knowledge in a field to inform health care decision-making and evidence-based policy. By employing meta-analysis—a statistical technique used to combine quantitative results of two or more studies [1]—they can address questions that otherwise could not be answered by individual studies. Systematic reviews can also inform future research priorities and identify common problems in the design of included studies, which can inform design recommendations for future studies. However, the effort of undertaking a systematic review is wasted if authors do not report completely what methods they used and what results they found [2]. Incomplete reporting poses a barrier to the inclusion of reviews in clinical practice guidelines and overviews of systematic reviews and to updating and replication of systematic reviews. Furthermore, without providing a clear description of the interventions delivered in studies included in a systematic review, healthcare providers will likely struggle to understand how best to deliver effective interventions in practice [3]. Incomplete reporting thus represents one form of research waste where previous research investment cannot be leveraged upon [2].

Reporting guidelines are designed to address incomplete reporting of research. They typically comprise a checklist or explanatory text to guide authors in reporting for a specific study design and are developed using a range of methods (e.g. surveys gathering feedback on the relative importance of potential items) [4]. The most highly cited reporting guideline—with > 100,000 citations—is the original PRISMA statement for reporting systematic reviews of health interventions (‘PRISMA 2009’) [5]. There are 14 extensions of the PRISMA 2009 statement [6–19] which provide reporting guidance for specific stages of systematic reviews (e.g. protocol [6]), for particular synthesis methods (e.g. network meta-analysis [7]), or for systematic reviews of particular study designs (e.g. diagnostic test accuracy [8]). Several new PRISMA extensions are currently being prepared [20]. Some of these extensions in development are being prepared as extensions of the PRISMA 2020 statement [21, 22], an update of PRISMA 2009 which includes new reporting guidance that reflects advances over the last decade in methods to identify, select, appraise and synthesise studies.

As the number of PRISMA extensions has grown, so too has the burden on authors and journals, who might need to use multiple guidelines for a particular review. For example, authors undertaking a systematic review of the effects of a complex intervention, in which network meta-analysis (NMA) of individual participant data (IPD) was conducted, would need to consult four guidelines (PRISMA 2020 [21, 22], PRISMA Complex Interventions [10], PRISMA NMA [7] and PRISMA IPD [12]), translating to 120 pages of guidance. Furthermore, there are important differences across these four guidelines in the wording of particular items that authors need to reconcile, which can lead to inconsistency in how an item is interpreted and reported against. These differences are unsurprising because PRISMA extensions have been developed independently over time by different teams. A harmonised approach for ensuring that recommendations, wording and layout are consistent across PRISMA guidelines should facilitate usability and uptake, in turn increasing the value of systematic review reports and their ensuing use in decision-making.

Greater usability and uptake of the PRISMA reporting guidelines might be further realised through technology. The current passive strategy of implementing the PRISMA reporting guidelines, which has primarily consisted of publication in journals [23], has not fully brought about needed improvements in the accuracy and completeness of reporting. For example, our investigation of systematic reviews published in 2020 revealed many items are still reported in less than 50% of systematic reviews, even in those that claim to follow PRISMA [24]. Several active strategies involving the use of technology have been developed to implement another reporting guideline—the CONSORT statement for randomised trials [25]—and evaluations of these strategies show promising results. For example, medical students randomly allocated to use an online writing tool with guidance and examples from the CONSORT statement embedded within it (‘COBWEB’) produced a more complete report of a randomised trial than students assigned to a PDF copy of the CONSORT statement [26]. Also, early career researchers using an online CONSORT-based peer review tool (‘COBPeer’) were more likely to detect inadequate reporting in trial reports than the original peer reviewers of the reports [27]. However, no equivalent active strategies have been developed to implement any of the PRISMA reporting guidelines.

### Objectives

Our aim is to use novel methods to enable more efficient and effective translation of PRISMA reporting guidelines into practice, thereby increasing the quality of systematic reviews which are critical to public health and policy decisions. We will:

1. Establish a working group who will develop a unified PRISMA statement that harmonises content across the main PRISMA guideline and its extensions.
2. Develop a web application that streamlines the process of reporting systematic reviews (PRISMA-Web) and evaluate its impact on authors’ reporting.
3. Develop a web application that helps peer reviewers appraise systematic review manuscripts (PRISMA-Peer) and evaluate its impact on peer reviewers’ detection of incomplete reporting.

## Methods

### Overview

The PRISMATIC (‘PRISMA, Technology, and Implementation to enhance reporting Completeness’) project comprises a suite of studies to address the three research objectives. Here, we outline the planned methods of each study, indicating where we plan to publish separate protocols with further details. We will declare any deviations from our planned methods in reports presenting the results of the research. To allow others to verify our findings, we will make the data and analytic code used to generate results publicly accessible via the Open Science Framework repository when reports of the results are published.

### Study 1: Development of a unified PRISMA statement

The objective of this study is to develop a reporting guideline that harmonises content across the PRISMA 2020 statement and several PRISMA extensions (i.e. a unified PRISMA statement). We will establish a ‘unified PRISMA Working Group’ to undertake this work. We (the authors of this protocol) will send an email to corresponding authors of PRISMA reporting guidelines and other aligned guidelines that are published, complete but not yet published, in development, or proposed but not started, inviting them to join the working group or to nominate a suitable alternative. We will also invite other stakeholders (e.g. editors of journals that frequently publish systematic reviews or representatives of funding bodies that frequently fund systematic reviews) to join the working group. We anticipate most members will be working in the discipline of health and medical research but will place no restrictions on the discipline.

We consider it useful to ultimately harmonise the following guidelines:

1. Anything identified as a PRISMA reporting guideline
2. Other reporting guidelines for evidence synthesis, including those for reporting:
  - A particular type of evidence synthesis (e.g. systematic review, scoping review, overview of systematic reviews)
  - A particular type of question (e.g. intervention effects, diagnostic test accuracy)
  - A particular synthesis method (e.g. network meta-analysis, synthesis without meta-analysis)
  - Synthesis of a particular type of data (e.g. individual participant data, qualitative data)
  - Synthesis of a particular population (e.g. children), intervention (e.g. non-pharmacological) or outcome (e.g. harms)

However, we will prioritise for the current project harmonisation of the following guidelines:

- Anything identified as a PRISMA reporting guideline for systematic reviews of the effects of health interventions, which is published or completed and made available to us before June 30, 2023
- Other reporting guidelines for systematic reviews of health interventions that are explicitly aligned with or borrow heavily from the original PRISMA statement or the PRISMA 2020 statement

We will identify relevant reporting guidelines by retrieving the full text of all reporting guidelines classified under the ‘Systematic reviews/Meta-analyses/Reviews/HTA/Overviews’ category on the comprehensive searchable database of reporting guidelines maintained by the EQUATOR (‘Enhancing the QUAlity and Transparency Of health Research’) Network. Two investigators will independently screen the full text of each guideline retrieved against the eligibility criteria, and any discrepancies in screening judgements will be resolved via discussion or adjudication by another investigator. For each guideline included, we will retrieve the checklist of recommended reporting items and, if available, the accompanying explanation and elaboration paper; the latter typically provides more detailed recommendations and examples of complete reporting. Two investigators will independently extract all reporting recommendations from each reporting guideline into a Microsoft Excel file. Both investigators will then use NVivo software to classify each reporting recommendation using a code reflecting the content of the recommendation (starting with a pre-specified coding framework, which we will revise where necessary). Both investigators will also analyse the wording of recommendations across the guidelines and record which are *common* (use identical wording), which are *similar* (address the same concept but with different wording) and which are *unique* to the particular extension (see Table 1 for examples). Any discrepancies in classifications of text will be resolved via discussion or adjudication by another investigator. We will then send the classifications to the corresponding authors (or their nominees) of each guideline and ask them to confirm whether they agree with our interpretations or suggest an alternative classification.

**Table 1:** Examples of classifying PRISMA items

**Item common to all PRISMA extensions:** ‘Describe the rationale for the review in the context of what is already known’
**Item worded similarly, with important differences, across PRISMA extensions:** PRISMA Harms recommends, ‘Present full *electronic* search strategy *for at least one database*, including any limits used, *such that it could be repeated*’ [15]. PRISMA 2020 recommends, ‘Present the full search strategies *for all databases, registers and websites*, including any *filters and *limits used’ [21]
**Item unique to a PRISMA extension:** Only PRISMA NMA recommends, ‘Describe the statistical methods used to evaluate the agreement of direct and indirect evidence in the treatment network(s) studied’ [7]

We will then convene a series of virtual consensus meetings with the unified PRISMA Working Group to develop the unified PRISMA statement—that is, a minimum set of items that can be adopted across PRISMA guidelines, regardless of the characteristics and methods of a review. This harmonised set of items can then be combined in customisable checklists with items that are unique to each extension that is relevant to a particular review. The proposed structure of the unified PRISMA statement will mirror that of the PRISMA 2020 statement, that is, a list of reporting items corresponding to different sections of a systematic review manuscript, along with bullet points under each item that detail the reporting recommendations [21, 22]. At each consensus meeting, relevant results of the content analysis will be presented, along with proposals for harmonised wording of items as drafted by the study investigators. Between the meetings, the corresponding authors of each reporting guideline will seek feedback from the co-authors of their guideline on the proposals suggested at the meetings. Any edits suggested by guideline co-authors will then be discussed by all members of the unified PRISMA Working Group.

Following the consensus meetings, we will prepare a draft of the unified PRISMA statement and circulate it to the unified PRISMA Working Group members for feedback and revise it until there is consensus on the content. We will then conduct semi-structured interviews with authors and journal editors from various countries, to ensure users are interpreting the items correctly, and identify any problematic items that need revision. We will audio record and transcribe the interviews verbatim, which one investigator will code deductively, and another will verify. Once the unified PRISMA statement is finalised in response to data from the qualitative interviews, we will disseminate it freely online on the PRISMA statement website (http://prisma-statement.org/). The unified statement will be the first of its kind, as there has been no prior attempt to harmonise existing reporting guidelines for any study design.

### Study 2a: Development and pilot testing of the PRISMA-Web app

**Fig. 1:**
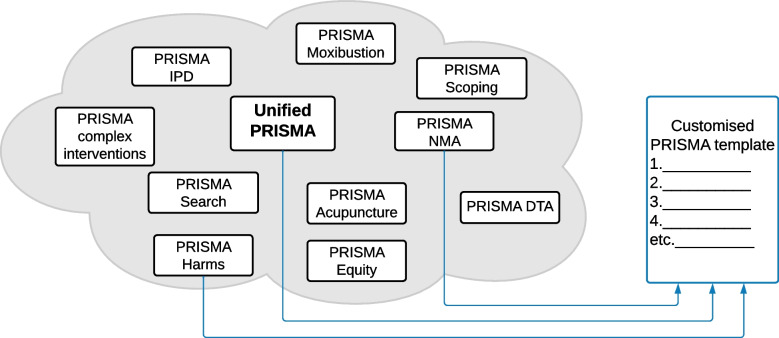
Model of the PRISMA-Web app. In the example depicted, a customised checklist and template for a systematic review with network meta-analysis of adverse events associated with COVID-19 vaccines are created. The items are drawn from three statements—the unified PRISMA statement, PRISMA Harms extension [15] and PRISMA NMA extension [7]. Adapted from Hopewell et al. [28]

The objective of this study is to develop and pilot test a freely accessible, open-source web application that generates a reporting template and checklist customised to the characteristics and methods of a systematic review (‘PRISMA-Web app’). After users answer a set of questions about their systematic review (e.g. ‘Was network meta-analysis conducted?’, ‘Did you include individual participant data?’), the app will select relevant items from the unified PRISMA statement and all relevant PRISMA extensions and construct a customised template in which authors can write the background, methods, results and discussion sections of their systematic review (see Fig. 1 for an example). Relevant PRISMA items and links to accompanying reporting guidance (e.g. elements, explanations and elaborations), educational videos and exemplars will appear throughout the template to guide reporting. Adapting the WebCONSORT [28] concept to systematic reviews, the app will also generate a fillable checklist customised to the characteristics and methods of the systematic review, for authors to complete at the stage of submitting a manuscript for publication (in which they can indicate the location in their manuscript where each PRISMA item is addressed).

Two investigators will conduct testing to ensure that the correct set of PRISMA items are extracted from the server for different types of systematic review. To do this, we will work through the list of all possible scenarios of systematic review that was used to create the app. The scenarios will cover all combinations of PRISMA extensions (e.g. unified PRISMA plus PRISMA NMA; unified PRISMA plus PRISMA NMA plus PRISMA IPD, etc.). We will then enter the characteristics and methods corresponding to each of these scenarios into the PRISMA-Web app and verify that all the correct items have been extracted from the server and appear in the customised template and checklist.

After verifying that the app functions as expected, a qualitative researcher will conduct user testing following methods developed by Rosenbaum et al. [29–32], in which review authors will be asked to ‘think-aloud’ as they navigate through the beta version of the PRISMA-Web app, to gather feedback on its usability and identify where refinements are necessary. Interviews will be held remotely using video meeting software (Zoom). During the testing, we will invite participants to navigate through the PRISMA-Web app, first giving general impressions as they explore the app freely, and then using the app to generate a template for a scenario relevant to their work. Participants will be encouraged to provide honest impressions, including positive and negative feedback, things they find difficult and easy to use, overall usefulness and suggestions for improvement. We will video record interviews (with shared screen view to observe problems with navigation) and an observer will take notes. One investigator will code and analyse data, and then results will be discussed with members of the app development team to identify solutions to problems and improvements.

### Study 2b: Randomised trial evaluating PRISMA-Web app

The objective of this study is to evaluate whether the use of the PRISMA-Web app improves the completeness of authors’ reporting of systematic reviews when compared with usual practice. We will do this by conducting an individually randomised parallel-group trial. Detailed methods of the randomised trial will be published in a separate trial protocol. Briefly, authors who are commencing a write-up of their systematic review of health research will be eligible to participate. We will randomly assign participants in a 1:1 ratio to write their review using the customised template generated by the PRISMA-Web app (intervention) or using a blank online form with links to all PRISMA guidelines (control). We will contact all participants 10 months post-randomisation to obtain a copy of their systematic review manuscript, regardless of whether it has been submitted for publication. Two investigators who are blind to group allocation will independently assess whether each relevant PRISMA item is ‘completely reported’ or ‘partially/not reported’. The primary outcome will be the proportion of items completely reported per systematic review.

### Study 3a: Development and pilot testing of the PRISMA-Peer app

The objective of this study is to develop and pilot test a freely accessible, open-source web application that can be used by peer reviewers when evaluating a systematic review manuscript (‘PRISMA-Peer app’). The app expands the COBPeer [27] concept in that it will generate a *customisable* list of PRISMA items for peer reviewers to assess, depending on the characteristics and methods of the systematic review. It will require users to record (by ticking ‘yes/no’ boxes) whether each relevant PRISMA item is reported completely in a manuscript and, like the PRISMA-Web app, will embed prompts, detailed guidance and examples throughout to help them make such judgements. Once each item is rated as reported or not, the app will automatically generate a standardised and individualised peer-review report detailing what information is missing from the manuscript, which could be provided to authors along with any other comments peer reviewers might have on the novelty, design and interpretation of the review. We will recruit a convenience sample of journal editors and peer reviewers to pilot test a beta version of the app and a qualitative researcher will conduct user testing using the think-aloud approach adopted in Study 2a to gather feedback on its usability and identify any necessary refinements.

### Study 3b: Cross-sectional diagnostic study evaluating PRISMA-Peer app

The objective of this study is to evaluate whether the PRISMA-Peer app improves peer reviewers’ ability to detect incomplete reporting in systematic reviews**.** We will do this by conducting a cross-sectional diagnostic study using an approach adopted previously when evaluating the COBPeer tool for randomised trial reports [27]. Detailed methods of the diagnostic accuracy study will be published in a separate study protocol. Briefly, we will assemble a sample of systematic reviews published in journals for which peer review reports are publicly available (e.g. in *BMJ*, *BMJ Open*) and compare the number of items detected as completely reported as assessed by the following: (i) volunteers who re-review the manuscript using the PRISMA-Peer app; (ii) the usual peer review process (i.e. what was documented in the original peer review report); and (iii) two assessors with expertise in systematic review reporting not using the app (reference standard). The primary outcome will be the proportion of items accurately classified as being incompletely reported per manuscript by the participants using the PRISMA-Peer app and by the original peer reviewers. Secondary outcomes will be the sensitivity, specificity, positive likelihood ratio and negative likelihood ratio for participants using the PRISMA-Peer app and the original peer reviewers to accurately detect the items as incompletely reported. For all outcomes, accuracy will be determined with regard to the reference standard.

## Discussion

The PRISMATIC project will develop and evaluate the effectiveness of novel strategies designed to improve the implementation of PRISMA reporting guidelines into practice. Our research will add to the few studies evaluating interventions designed to improve the uptake of PRISMA, which have been limited to evaluating whether a journal mandate or encouragement to submit a PRISMA checklist alongside an author’s systematic review increases the completeness of reporting [33, 34]. If found to be successful, the methods we develop could potentially be adapted to reporting guidelines for other study designs. The project also provides an opportunity to establish a working group to facilitate closer collaboration between teams during the development and updating of PRISMA guidelines. The creation of such a working group has been found to be useful in the development of other methodological guidance (e.g. GRADE) [35].

The production of PRISMA guidelines by different teams at different times has inadvertently resulted in confusion for the many authors who conduct reviews for which multiple PRISMA extensions are relevant. Our proposal to harmonise content across existing extensions and develop a unified PRISMA statement should simplify the process for authors of systematic reviews, removing the need to make sense of inconsistent guidance and determine which recommendations to follow. The unified PRISMA statement will also provide a foundation for updates to current extensions (e.g. for systematic reviews with NMA) and for the development of new extensions for which no guidance is currently available (e.g. for systematic reviews of prevalence studies, and mixed-methods systematic reviews).

The passive, and ubiquitous, implementation strategy for PRISMA reporting guidelines has been shown to have a limited effect on systematic reviewers’ reporting [24, 36]. A key reason for this is that authors typically consult PRISMA and its extensions at the point of submitting their review manuscript to a journal. At this late stage, they might be resistant to revising what they have written to comply with PRISMA. Adapting an approach found to be successful for writing randomised trial reports [26], we plan to develop a web application—PRISMA-Web—that prompts complete and accurate reporting of systematic reviews much earlier in the writing process. The use of PRISMA-Web when commencing writing should lessen the need for extensive revisions during the peer-review process, thus saving time. By helping authors report their review more completely and accurately, the PRISMA-Web app should enable the production of systematic reviews that better meet the needs of stakeholders.

The peer review process exists to help ensure that research papers submitted for publication are vetted for rigour, relevance and completeness before they are published. Despite being widely perceived as a valuable endeavour, surprisingly little research has been done to evaluate how effective peer review is and how it can be improved [37]. Peer reviewers of systematic reviews currently need to perform various tasks, often manually and with little or no targeted guidance, including assessing the novelty and significance of the review question, determining whether the review methods are sound and the results interpreted appropriately and evaluating whether all aspects have been reported completely (i.e. in line with recommended PRISMA guidance) [38]. Our proposed research challenges the notion that peer reviewers can do all these tasks effectively and efficiently. We will produce, for the first time, technology that aids peer reviewers of systematic reviews. If shown to increase accuracy, a PRISMA-Peer assessment could become a standard component of editorial management systems worldwide, with the app used by trained early career researchers or editorial managers. This would help cover the critical shortage of peer reviewers with methodological expertise and reduce the workload for peer reviewers with clinical expertise.

## Conclusion

We anticipate that the novel guidance and web-based apps developed throughout the project will substantively enhance the completeness of reporting of systematic reviews of health evidence. This should ultimately benefit the many users who rely on systematic reviews to inform practices and policies designed to improve health outcomes.

## Data Availability

No data are associated with the article.
